# Selection of Apoptotic Cell Specific Human Antibodies from Adult Bone Marrow

**DOI:** 10.1371/journal.pone.0095999

**Published:** 2014-04-23

**Authors:** Caroline Grönwall, Edgar D. Charles, Lynn B. Dustin, Christoph Rader, Gregg J. Silverman

**Affiliations:** 1 School of Medicine, New York University, New York, New York, United States of America; 2 Laboratory of Virology and Infectious Disease, Rockefeller University, New York, New York, United States of America; 3 University of Oxford, Oxford, United Kingdom; 4 Department of Cancer Biology and Department of Molecular Therapeutics, The Scripps Research Institute, Jupiter, Florida, United States of America; Cordelier Research Center, INSERMU872-Team16, France

## Abstract

Autoreactive antibodies that recognize neo-determinants on apoptotic cells in mice have been proposed to have protective, homeostatic and immunoregulatory properties, although our knowledge about the equivalent antibodies in humans has been much more limited. In the current study, human monoclonal antibodies with binding specificity for apoptotic cells were isolated from the bone marrow of healthy adults using phage display technology. These antibodies were shown to recognize phosphorylcholine (PC)-associated neo-determinants. Interestingly, three of the four identified apoptotic cell-specific antibody clones were encoded by VH3 region rearrangements with germline or nearly germline configuration without evidence of somatic hypermutation. Importantly, the different identified antibody clones had diverse heavy chain CDR3 and deduced binding surfaces as suggested by structure modeling. This may suggest a potentially great heterogeneity in human antibodies recognizing PC-related epitopes on apoptotic cells. To re-construct the postulated structural format of the parental anti-PC antibody, the dominant clone was also expressed as a recombinant human polymeric IgM, which revealed a substantially increased binding reactivity, with dose-dependent and antigen-inhibitable binding of apoptotic cells. Our findings may have implication for improved prognostic testing and therapeutic interventions in human inflammatory disease.

## Introduction

Within the adaptive immune system B-lymphocyte clonal selection enhances defense from exogenous threats. Yet this strictly regulated process for immune cellular selection also incorporates clonal deletion (i.e., negative selection) of self-reactive clones that may otherwise contribute to pathogenic autoimmunity [1]. Nonetheless, some types of autoreactive clones are common physiologic components of the immune system that recurrently arise during early immune development, and these are postulated to contribute to homeostasis through specialized immune functions [2]–[6].

One of the most fundamental housekeeping functions of the immune system involves the clearance of dead and dying cells as part of physiologic cell turn-over and tissue repair mechanisms. Each day more than 100 billion cells die by apoptosis and these must be rapidly eliminated to prevent the release of autoantigens and danger-associated signals that otherwise can mediate inflammatory responses. To maintain homeostasis as well as limit and resolve inflammation, there are layered inhibitory mechanisms that are intertwined with the recognition and clearance of damaged host cells. These pathways may be reinforced by autoreactive antibodies that specifically recognize apoptotic cells (ACs) [7].

There is now mounting evidence of spontaneously arising antibody responses, which by specific recognition of oxidation-associated neo-determinants on ACs, can discriminate dying from healthy cells to facilitate a number of physiologic roles and regulatory functions (reviewed in[6]). A range of apoptosis-associated epitopes have been reported that are recognized by B-cell clones and their antibody products. Highly prominent, the phosphorylcholine (PC) head group, that is ubiquitously expressed in neutral cell membrane phospholipids, can be part of apoptotic-associated neo-determinants. While PC-containing moieties are sequestered in healthy cells, during programmed cell death these can undergo chemical modifications that result in exposure and accessibility for antibody binding [8]–[10].

Human anti-PC antibodies are prevalent in the bloodstream in both healthy individuals and during certain disease states [11]–[13], and levels of IgM anti-PC antibodies are reported to directly correlate with serum antibody binding to membrane neo-determinants of ACs [14]. Moreover, high levels of IgM anti-PC have been correlated with protection from atherosclerotic cardiovascular events, as well as lower overall disease activity in SLE patients (reviewed in [6],[15]. However, there is currently little known about the molecular and immunogenetic features of AC-specific human antibodies.

To isolate human antibody clones that recognize ACs, we designed a strategy that uses proven phage display antibody technology in which there is a physical linkage between antigen-binding particles and the encoding somatically rearranged antibody genes [16]. Based on the assumption that anti-AC antibodies are highly represented in the human immune system, we sought to select antibodies from a large library generated from human bone marrow, which contains a cellular immune record of an individual's life-time antigenic experiences [17]. Our findings suggest that diverse structural strategies can be utilized in the formation of AC-specific human antibodies that target PC-containing neo-determinants.

## Methods

### Phage display library

A human naïve antibody Fab-fragment phage display library was generated with bone marrow from six healthy adult volunteers as previously reported [18], [19] by adapting established methods [20]. Briefly, a total of 186 different Vκ, Vλ, and V_H_ RT-PCR amplimers were assembled by overlap extension PCR into Vκ-Cκ-V_H_ and Vλ-Cλ-VH cassettes, which were then cloned into the pC3C phagemid vector [18], [19]. After transformation of XL1-Blue *E. coli* (Stratagene) and subsequent infection with the VSCSM13 helper phage (Stratagene), a pooled phage library was generated that was estimated to contain 1.5×10^9^ independent Fab clones. For expression and purification of Fab-(His)_6_ fusion proteins (hereafter termed Fab), selected clones were subcloned into the pC3C-His vector [21] for protein expression in the TOP10 *E. coli* strain. A control tetanus toxoid specific (TT) antibody [22] was expressed as a Fab protein using the pComb3X vector [20] (provided by C. F. Barbas III, The Scripps Research Institute).

### Phage display selection

The human bone marrow Fab phagemid library was subjected to five sequential rounds of selection and in vitro expansion, under conditions described in Table 1. For PC-ligand mediated selection, high valency PC16-BSA (Biosearch Technologies) was biotinylated using EZ-link sulfo-NHS-LC-biotin (Thermo Scientific) and the resulting complexes of phage-Fab with PC-antigen were captured with aliquots of paramagnetic beads (MyOne streptavidin T1, Invitrogen). To generate ACs, Jurkat cells were cultured for 16 hrs with 40 µM etoposide (Sigma Aldrich) in complete RPMI media, and cells were then washed three times in cold PBS and resuspended at 25×10^6^ cells/ml. One ml aliquots of AC suspensions were then biotinylated by incubation for 30 min with 1 mg sulfo-NHS-LC-biotin, the reaction was subsequently quenched with three washes of 100 mM glycine in PBS. For selection on ACs, aliquots of 1.25×10^6^ biotin-tagged apoptotic Jurkat cells were mixed with 0.5 mg of streptavidin coated paramagnetic beads, then washed prior to incubation with an aliquot of the phage library. To avoid non-specific binding, all tubes and beads were first blocked with PBS containing 0.1% Tween 20 and 5% BSA (5% T-PBSB). In addition, to further reduce the representation of unwanted non-specific binders, before each round of panning the aliquots of phage libraries were first subjected to one round of negative selection by pre-adsorption for 30 minutes with streptavidin coated beads that had been incubated with the irrelevant ligand, azobenzene arsonate (ABA)-BSA-biotin conjugate (Biosearch Technologies).

**Table 1:** Strategy for selection of Fab-expressing phagemid clones.

Selection Cycle	Target ligand	Concentration of protein ligand	Number of washes
**1**	PC16-BSA	100 nM	3
**2**	PC16-BSA	25 nM	5
**3**	PC16-BSA	25 nM	8
**4**	1.25×10^6^ ACs	-	6
**5**	1.25×10^6^ ACs	-	8

Selections by the experimental PC-antigenic ligand, or with beads coated with ACs, were carried out in 1 ml aliquots 3% T-PBSB with continuous rotation for 2 hrs at room temperature. Solution phase ligand-phage complexes were captured by 30 min incubation with beads. Thereafter, beads with bound phage were washed, as indicated (Table 1), and bound phage were eluted with 200 mM glycine-HCl pH 2.2, followed by immediate neutralization with 1 M Tris-HCl, and infection of XL-1 blue *E. coli* in logarithmic growth phase. To generate the phage form of the library for the next round of selection, these bacterial cultures were then rescued by infection with VSCSM13 helper phage, amplified overnight, purified and then precipitated with PEG/NaCl. The stringency of the selection process was increased in each of the sequential selection cycles by lowering the concentration of the selecting ligand, and by increasing the number of washes, according to Table 1.

The outcome of each round of selection was monitored by titration of the phage stocks before and after elution, using serial dilutions of eluted phage that were used to infect bacteria that were spread onto agar plates containing 100 µg/ml ampicillin. From each cycle, the binding activity of the input phage pools for PC-BSA was evaluated by standard ELISA, with detection using horse radish peroxidase (HRP) conjugated anti-M13 phage mAb (GE Healthcare).

### Post-selection screening

Individual clones from selection round five were randomly picked and used to separately inoculate 1-ml aliquots of super broth (SB) media with carbenicillin (100 µg/ml), tetracycline (10 µg/ml), and 2% glucose in 96-well plates and grown over night at 37°C with shaking. Cultures were then inoculated into fresh media without glucose and after 4 hrs expression was induced by addition of 1 mM IPTG, with over night culture at 37°C. The following day, cells in cultures were spun down and Fab-containing supernatants were evaluated in ELISA. Briefly, these supernatants were diluted with an equal volume of 3% BSA with 0.2% Tween 20 in PBS, and were screened in parallel for Fab expression, binding to PC4-BSA (Biosearch Technologies) and the control antigen, malondialdehyde (MDA)-BSA (Academy Biomedical Co) using a standard ELISA protocol, with detection with HRP-conjugated goat anti-human Fab (Jackson ImmunoResearch). Clones with high PC-binding and Fab expression, and no detectable MDA-BSA binding, were individually re-amplified from glycerol stocks. Plasmids were then purified with miniprep columns (Qiagen) and VH and VL region encoding DNA inserts were sequenced using specific primers (VL-Seq 5′GATAACAATTGAATTCAGGAG3′; VH-seq 5′TGAGTTCCACGACACCGT3′). DNA sequences were compared to known germline sequences using Ig-BLAST [23].

The unique Fab clones, p2–7, p2–20, p2–31 and p2–81, were also prepared as phage in solution from 25-ml cultures. Overnight cultures were grown in SB media with 2% glucose, carbenicillin (100 µg/ml) and tetracycline (10 µg/ml), and the following day inoculated in larger volumes and grown 4 hrs until logarithmic growth phase. Bacteria were centrifuged and then dispersed into new SB media supplemented with carbenicillin, tetracycline and 0.1 mM IPTG. Helper phage were then added to the cultures, followed by addition of kanamycin (70 µg/ml) 2 hrs later and culturing overnight at 37°C, with shaking. Phage were precipitated with PEG/NaCl. In all experiments fresh phage stocks were used the same day. The preparations gave approximately equal concentrations of phage particles. Two randomly picked clones from the unselected library were prepared as controls (denoted C1, C2).

### Expression and purification of Fab antibody fragments

Selected clones were subcloned into the pC3C-His vector using the Sfi I digestion site (New England Biolabs) with T4 ligation (Gibco) and expressed as hexa-histidine, Fab-(His)_6_, fusion proteins in TOP10 *E. coli*. Overnight bacterial cultures were used to inoculate 75 ml of SB media supplemented with carbenicillin and glucose (2%) and grown with shaking until logarithmic growth phase (OD 0.5-0.6) was attained. Glucose-containing media was then removed by centrifugation, and bacteria dispersed into 150 ml of fresh SB media with carbenicillin and grown for an additional 30–45 min. Protein expression was induced by 0.5 mM IPTG and cultures were grown overnight at 24°C with shaking. Bacteria were harvested by centrifugation and periplasmic fractions prepared by incubation with osmotic shock buffer (20% sucrose in 100 mM Tris-HCl, 1 mM EDTA, pH 7.4) 45 min on ice, followed by 30 min centrifugation at 15,000 *g*. Protein supernatants were buffer exchanged to phosphate buffer (10 mM Na_2_HPO_4_, 10 mM NaH_2_PO_4_, 0.5 M NaCl, pH 7.4) with dialysis using 20,000 MWCO Slide-a-lyzer cassettes (Thermo Scientific) and Fab proteins were purified by immobilized metal ion affinity chromatography (IMAC) using His-Gravitrap (GE Healthcare) columns following the manufacture's recommendations using 20 mM imidazole in the binding buffer, 60 mM imidazole in the washing step, and subsequently the protein was eluted with buffer containing 500 mM imidazole. Eluted protein was then buffer exchanged and then concentrated with 30-kDa MWCO Amicon Ultra centrifugation units (Millipore). The concentration and purity of the Fab proteins were assessed by absorbance at 280 nm and SDS-PAGE analysis both with and without a reducing agent.

### Antigen binding by ELISA

Binding to PC or control antigens was examined by standard ELISA, with wells coated overnight with 3 µg/ml of the antigen in PBS, blocking with 3% BSA in PBS, and detecting with HRP-conjugated goat anti-human Fab (Jackson ImmunoResearch) followed by 3,3′,5,5′-tetramethylbenzidine (TMB) substrate. For inhibition studies, aliquots of the antibodies were separately mixed with an antigen at indicated concentrations before adding to microtiter wells for immunoassay.

### IgM expression system

To express recombinant IgM, we used the pIgM, and Igκ or Igλ vectors [24], with expression in HEK293T cells that stably express the Ig J-chain, as previously described [24]. The IgM clone, mGO53, which expresses VH4-39, Vκ3-15 gene rearrangements, was used as a control antibody without known antigen binding activity [24], [25]. The PC-selected clone, p2–20, was further subcloned into the pIgM and Igλ expression vectors, as previously described [24]. For this purpose, the p2–20 VH and VL encoding genes were separately amplified by specific primers that added 5′ Age I and 3′ Xho I restriction sites, and PCR products were purified by QIAExII gel purification (Qiagen), cleaved by Age I and Xho I (New England Biolabs), and then repurified with Qiaquick purification kit (Qiagen) prior to ligation into cleaved and purified H and L chain vectors, respectively, with T4 ligase. Plasmids for transfections were prepared using Purelink Maxiprep kit (Invitrogen) following the manufacturer's instructions. HEK293T cells expressing a stably transfected human J-chain [24] were cultured until 80% confluent in DMEM with 10% low IgG FBS with 1 mM sodium pyruvate, penicillin, streptomycin, glutamine (Invitrogen). Shortly before transfection the media was changed to DMEM with 1% Nutridoma supplement (Roche), 1 mM sodium pyruvate and glutamine without antibiotics. To transfect one 150-mm culture dish, equal amounts of immunoglobulin (Ig) H and L chain plasmids (28 µg per plasmid) were diluted into 3.37 ml of 100 mM saline, which was then mixed with 450 µl of buffer containing 0.45 mg/ml of the branched polyethylenimine (PEI, Sigma Aldrich), and after incubation for 10 min was thereafter added to the cells. After 6 hrs, the media was changed and then the IgM-containing supernatants were harvested every 24 hrs. The expression level of IgM was determined by standard μ-chain specific sandwich ELISA, coating with goat (Fab)'2 anti-human IgM (Jackson ImmunoResearch) and detecting with HRP conjugated mouse anti-human IgM (Southern Biotech).

To purify p2–20 IgM we used staphylococcal protein A (SpA) affinity chromatography for enrichment via the VH3-Fab binding site on SpA [26], [27]. Briefly, IgM from filtered cell supernatants were captured onto SpA resin (Repligen IPA-400HC) equilibrated with Gentle Ag/Ab binding buffer (Thermo Scientific) and eluted with Gentle Ag/Ab elution buffer (Thermo Scientific) at neutral pH and buffer exchanged by dialysis with 20 kDa MWCO Slide-a-Lyzer cassettes (Thermo Scientific) and concentrated with 100-kDa MWCO Amicon Ultra centrifugation units (Millipore).

### AC binding assays

Thymocytes harvested from 7-week old C57/B6 (from Jackson Laboratories) were cultured in RPMI media supplemented with 10% FBS, penicillin, streptomycin, and L-glutamine, at 2×10^6^ cells/ml. Apoptosis was induced by incubation with 1 µM dexamethasone (Sigma Aldrich) at 37°C for 4–12 hrs, as indicated. Phage preparations, purified Fab fragments, human umbilical cord (i.e., neonatal) plasma, or IgM supernatants, were separately incubated with ACs in 3% BSA in PBS for 1 hr on ice. Binding was detected with biotinylated goat anti-human Fab or goat anti-human IgM μ-chain specific antibodies (Jackson ImmunoResearch), followed by streptavidin conjugated to phycoerythrin (BD Biosciences). For inhibition studies, the samples, at the indicated concentrations, were mixed with antigens to a final concentration of 100 µg/ml, before incubation with the cells. Antibody binding specificity for ACs was assessed by co-staining with Annexin V (BD Biosciences) and 7AAD (BD Biosciences), according to the manufacturer's instructions. Flow cytometry data were acquired using a FACSCaliber instrument (BD Biosciences) and analyzed with FlowJo (TreeStar Inc.). Animals used in the study were housed in a NYU vivarium and supervised under an approved protocol by the NYU Institutional Animal Care and Use Committee (protocol number 130904-01).

### Biosensor analysis

Purified Fab proteins were evaluated for binding to PC-ligands by real-time bio-specific interaction analysis (Biacore 2000, GE Healthcare). Low density PC4-BSA conjugate (Biosearch Technologies) was immobilized by amine-coupling onto the carboxylated dextran layer of a CM5 chip, according to the manufacturer's recommendations, generating a 966 RU immobilized surface. The activity of the surface preparation was confirmed by injecting either 700 nM or 70 nM of the murine T15 IgG1 that has known PC-binding activity. Fab antibody fragments were screened for binding at 5 µg/ml (100 nM) in PBS with 0.005% Tween 20. All samples were run in duplicate and the surfaces were regenerated with 10 mM glycine pH 1.5 between injections. The regeneration process did not affect the stability of the surface. During these analyses, the signal derived from a reference surface, which had no immobilized ligand, was subtracted from the values obtained for responses for antibody binding to the surface coated with the ligand of interest. To confirm stability and concentration, in parallel the Fab proteins were also injected over a control surface with 760 RU of immobilized goat (Fab)'2 anti-human Fab (Jackson ImmunoResearch).

### Structure modeling of antibody variable regions

Models of the variable region encoded surfaces from the selected clones were generated with the Web Antibody Modeling, WAM tool [28], using dead-end elimination for the side-chains building and the VFF energy screen method. Illustrations of the structures were generated in PyMol (DeLano Scientific LLC). Electrostatic surfaces were calculated from PQR files (PDB2PQR [29]) using the Adaptive Poisson-Boltzmann Solver (APBS) software [30] with PyMol.

## Results

### Selection of antibodies from a pooled human bone marrow library

By phage display selection, human antibodies binding to ACs were isolated from a Fab library that was previously generated from the bone marrow of six healthy adult donors [18]. As shown in Table 1, to restrict the molecular binding specificity of the isolated antibody libraries, we first performed three sequential rounds of selection against PC-conjugated to BSA, and this was followed by two rounds of selection using apoptotic whole Jurkat cells.

**Figure 1:**
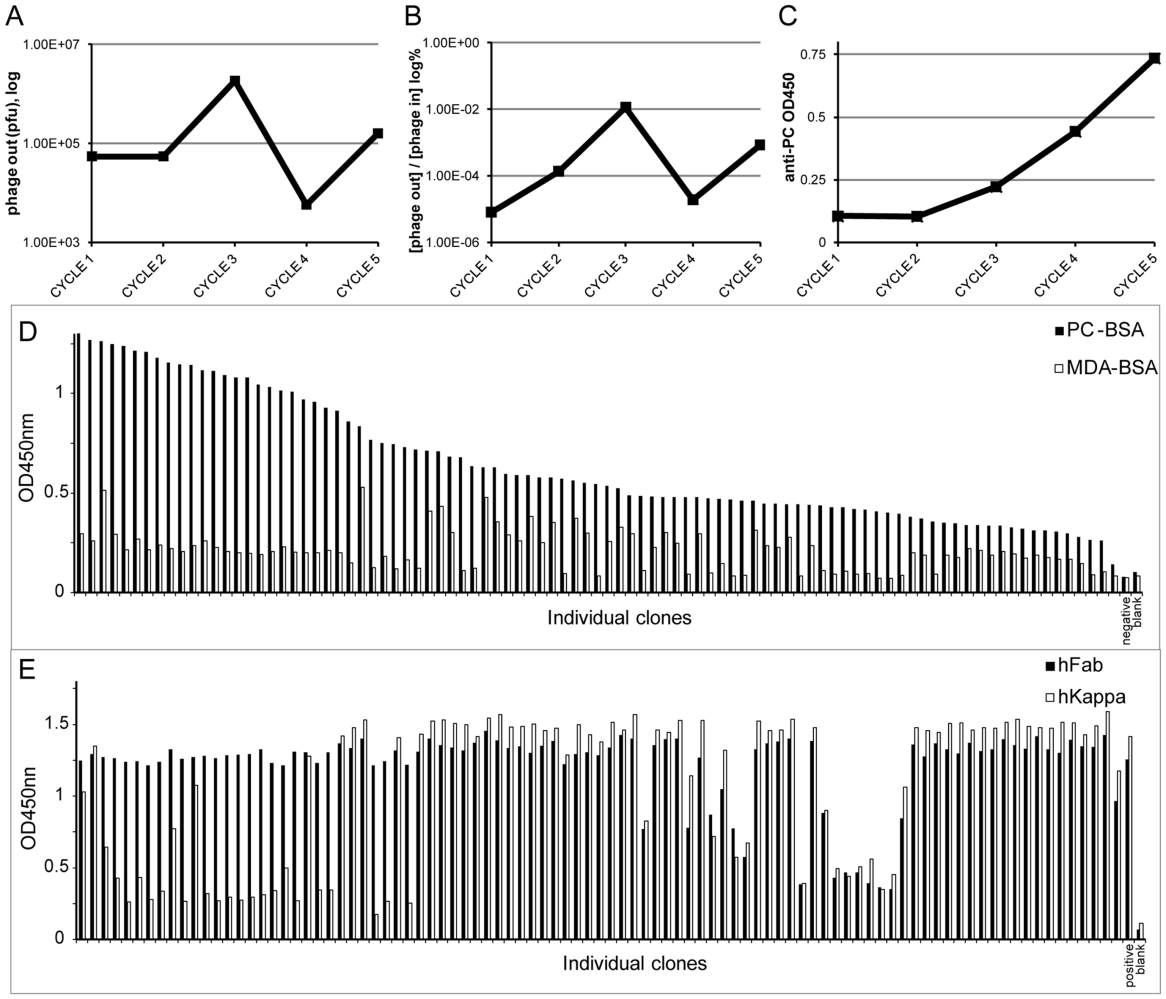
Phage display selection of human antibodies binding to PC and ACs. The first three rounds of panning used PC conjugated to BSA in solution, while cycle four and five amplified binding to whole apoptotic Jurkat cells. The amplification of binding phage in the selection is illustrated in (**A**) showing the titers of bound phage eluted in each selection cycle, and (**B**) showing an increase in the ratio of eluted phage (phage out) of the number of phage particles used going into each round (phage in) presented as % on a logarithmic scale and (**C**) showing the increase of PC-binding in ELISA of the starting phage libraries in each selection cycle at 1∶5 dilution and detection with anti-M13 phage. Randomly selected individually clones were cultured from selection round five and screened for antibody expression and PC-binding by ELISA using anti-Fab detection. **D.** Screening for PC-binding (black) compared to binding to the control antigen MDA-BSA (white). **E.** Screening for human Fab (black) and kappa light chain (white) expression. The data in both **D** and **E** were sorted based on PC reactivity from high to low. Bacterial supernatant without Fab-expressing phagemid was used as negative control and human IgG1 (κ) as positive control.

After each of the rounds of selection for PC-binding we found an amplification of eluted phage clones, and while the titer was lower when subsequently switching from soluble ligand to whole cells, there was a significant increase following the final round of selection on ACs (Figure 1A–B). Importantly, the level of PC-binding reactivity by the eluted Fab-bearing phage clones increased after each round of selection (Figure 1C), which could reflect an increase in the representation of phage clones and/or their intrinsic binding activity for PC-containing ligands.

To further characterize the selected antibodies, clones from the final round of selection were randomly picked, individually expanded and Fab in the crude supernatants were then analyzed. The vast majority (94%) were found to produce Fab that contained Ig VH and VL regions (Figure 1E). In parallel, we performed antigen-binding screenings to evaluate for interactions with oxidation-associated neo-determinants that are expressed on ACs. While almost every Fab-expressing clones had detectable PC-binding activity, a subset (25%) showed significantly higher PC reactivity while there was low/non-detectable binding to the control antigen, malondialdehyde (MDA)-BSA (Figure 1D). In addition, there was no binding activity detected for BSA alone (data not shown).

### Dominance within the library of selected anti-AC Fab clones

To evaluate the diversity within the selected library, Fab-expressing phagemid clones with high PC-binding activity were identified and the DNA sequences of the encoding antibody genes were determined. Among 28 clones, based on recurrent VH-VL gene expression our antibody gene sequence determinations identified four distinct antibody rearrangement clonotypes. Among these anti-PC clones, the most highly represented clone, p2–20, was present in 64% of sequence analyzed phagemids. We also found three additional, although less abundant, clonal sets; p2–7, p2–31 and p2–81 (Table 2 and 3). Importantly, there were no sequence variations among the copies of these recurrent distinct clones, and hence there was no evidence of intraclonal diversity.

**Table 2:** Selected PC-binding clones.

clone	% of screened*	VH genes (% germline identity)**	VH	DH	JH	VL	VL genes (% germline identity)***	VL	JL
**p2**–**7**	28%	(100%)	3–30	3–3	6	kappa	(91.2%)	VK3-20	JK3
**p2**–**20**	64%	(99.3%)	3–33	1–7	3	lambda	(95.6%)	VL1-44	JL3
**p2**–**81**	4%	(98.3%)	3–30	3–3	3	kappa	(93.3%)	VK1-5	JK1
**p2**–**31**	4%	(97.3%)	1–2	3–3	4	lambda	(92%)	VL1-47	JL1

* From sequencing screen of individual PC-binding clones after three rounds with PC-BSA conjugate and two rounds of phage display selection on whole AC.

** Compared to the VH, D and JH germline nucleotide gene combination with highest homology in the NCBI database using IgBLAST.

*** Compared to the VL, JL germline nucleotide gene combination with highest homology in the NCBI database using IgBLAST.

**Table 3:** VH sequences of PC-binding clones from phage display selection compared to the closest germline sequence.

	FWR1	CDR1	FWR2	CDR2	FWR3		D		J
**p2-7**	EVQLVESGGGVVQPGGSLRLSCAAS	GFTFSSYGMH	WVRQAPGKGLEWVA	FIRYDGSNKY	YADSVKGRFTISRDNSKNTLYLQMNSLRAEDTAVYYCAK	ADR	RFLEWLL	S	DYYGMDVWGQGTMVTVSS
VH3-30*02	*************************	**********	**************	**********	***************************************				
DH3-3*01							*******		
JH6*02									******************
**p2-20**	QMQLVQSGGGVVQPGRSLRLSCAAS	GFTFSSYGMH	WVRQAPGKGLEWVA	VIWYDGSNKY	YADSVKGRFTISRDNSKNTLYLQMNSLRAEDTAVYYCAR	SP	NWNY	LSVG	FDIWGQGTMVTVSS
VH3-33*01	*****E*******************	**********	**************	**********	***************************************				
DH1-7*01							****		
JH3*02									**************
**p2-81**	QVQLQESGGGVVQPGGSLRLSCAAS	GFTFSSYGMH	WVRQAPGKGLEWVA	FIRYDGSNKY	YADSAKGRFTISRDNSKNTLYLQMNSLRAEDTAVYYCAK	GS	LRFLEWLLY	RG	DAFDIWGQGTMVTVSS
VH3-30*02	****V********************	**********	**************	**********	****V**********************************				
DH3-3*01							*********		
JH3*02									****************
**p2-31**	KVQLVQSGAEVKKPGASVKVSCKAS	GYTFTDYYMH	WVRQAPGQGLEWMG	WINPNSGDTN	YAQKFQGRVTMTRDTSISKTYMELSRLRSDDTAMYYCAR	G	YFDFWSGY	RLMV	YWGQGTLVTVSS
VH1-2*02	*************************	*****G****	**************	*******G**	******************TA*************V*****				
DH3-3*01							*Y******		
JH4*02									************

* Identical to the closest known VH/DH/JH germline gene.

To evaluate the immunogenetic origins of the selected anti-PC clones, we identified the germline genes with the closest homology to these expressed gene rearrangements. Significantly, we found that the VH region of the p2–7 clone was encoded by a rearrangement with the germline configuration, without nucleotide substitution, of the VH3-30 gene. Furthermore, the most highly represented clone, p2–20, also had closest homology to a different member of the VH3 family, VH3-33, with only a single nucleotide substitution in framework 1 (FR1) subdomain, which encoded for a glutamine to glutamic acid replacement mutation in the codon for residue position 4, and a silent nucleotide substitution in the codon for residue 86 in FR3 subdomain. The third clone, p2–81, was also most homologous (98.3% identity) to the VH3-30 gene, with two replacement and three silent mutations, and therefore the p2–81 clone shared high homology with the VH rearrangement that encoded for the p2–7 clone. Notably, for all of these three VH3 encoded clones, the CDR1 and CDR2 of the VH regions were completely germline-encoded, as there were only nucleotide variations from the germline sequence in the framework regions that are not commonly directly involved in antigen recognition.

The fourth antibody clone identified, p2–31, was the most structurally and genetically diverse as it was most homologous to the VH1-2 member of the VH1 family, and it also displayed a somewhat higher level of somatically generated sequence variation with five replacement (one in CDR1, one in CDR2 and three in FR3) and two silent mutations (one in FR1 and one in FR3) (Table 2 and Table 3).

The closest germline DH and JH gene segments were also identified for the somatically generated HCDR3 of these clones (Table 2), and all antibody clones were also found to have N-insertions at the V-D and D-J junctions. In adults the average HCDR3 size is reported to be 31 nucleotides [31]. The clones p2–7 and p2–81 both had long HCDR3 lengths (51bp), while p2–20 and p2–31 had closer to the average HCDR3 length (36 bp and 39 bp, respectively). The HCDR3 of all clones had a negative or neutral net charge (p2–7, −3; p2–20, −1; p2–31, 0; p2–81, −1). The near-germline clones, p2–7, p2–20 and p2–81 had a similar number of aromatic amino acids (3–4 residues) while p2–31 had slightly more aromatic residues (6 residues). Furthermore, p2–7 and p2–81 with longer HCDR3 also had a higher number of hydrophobic residues (5 residues) compared to p2–20 and p2–31 (2 residues) (Table 2).

Analyses of the associated VL region rearrangements showed that p2–7 and p2–81 use kappa light chains, while p2–20 and p2–31 use lambda light chains. Notably, these light chains are 91.2% to 95.6% identical to the closest known VL germline gene segments (Table 2 and Table S1), suggesting that they had undergone greater levels of somatic diversification than the VH regions.

### Characterization of anti-PC purified Fab antibody fragments

**Figure 2:**
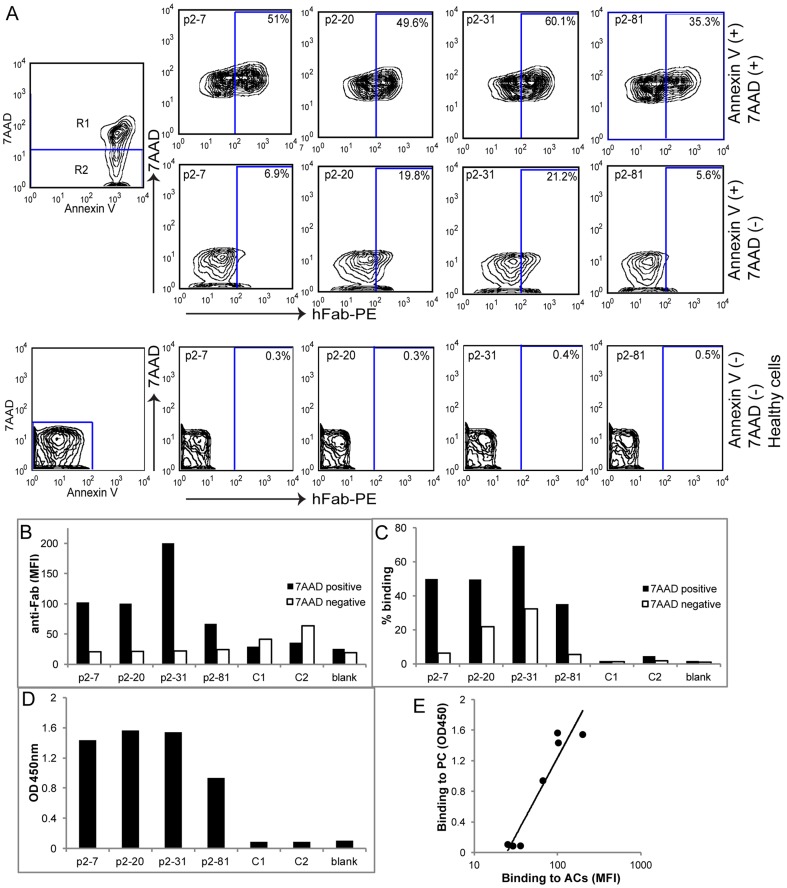
Selected unique anti-PC phage clones. Four unique VH clones were identified from sequencing individual PC-binding clones from selection round five. **A.** Flow cytometry screening of individual phage clones for binding to apoptotic mouse thymocytes treated with 1 µM dexamethasone for 13 hrs. The left panel is demonstrating the level of induced apoptosis, while the right panels are showing phage binding by detection of human Fab. The top right panel show binding to Annexin V (+) 7AAD (+) late apoptotic cells, the middle right panel show binding to Annexin V (+) 7AAD (−) early apoptotic cells and the bottom right panel show freshly isolated Annexin V (−) 7AAD (−) non-treated thymocytes. Binding was assessed with 30 µl phage stocks per 10^6^ cells. **B**. Mean fluorescence intensity (MFI) of phage binding to 7AAD (+) or 7AAD (−) ACs. **C.** Percentage binding of phage to 7AAD (+) or 7AAD (−) ACs obtained from the gating shown in A. **D**. Binding of the individual phage clones in ELISA at 1∶50 dilutions using anti-Fab detection. Control clones, C1 and C2, were randomly amplified from the unselected library. **E.** Correlation of phage stocks binding to PC-BSA in ELISA with binding to Annexin V (+) 7AAD (+) ACs by clones randomly picked from the original library (C1 and C2) and the PC-selected clones (p2–7, p2–20, p2–31, and p2–81; tested in duplicate assays).

Binding of these four selected clones of anti-PC antibodies was initially confirmed with immunoassays with Fab-displaying phage (Figure 1 & 2). In flow cytometry analysis, all four Fab-displaying phage were also found to bind to 7AAD (+) Annexin V (+) ACs, while none of the clones showed detectable binding to freshly isolated healthy thymocytes (Figure 2). Some binding was observed to 7AAD (−) Annexin V (+) that represent early stage apoptotic cells. The lower binding activity with early stage apoptotic cells, compared to late stage 7AAD (+), cells, may in part reflect the antigen density of neo-determinants expressed during the progression of apoptosis. Randomly picked clones from the unselected library showed neither detectable binding to apoptotic nor to healthy cells (Figure 2B, C and D).

**Figure 3:**
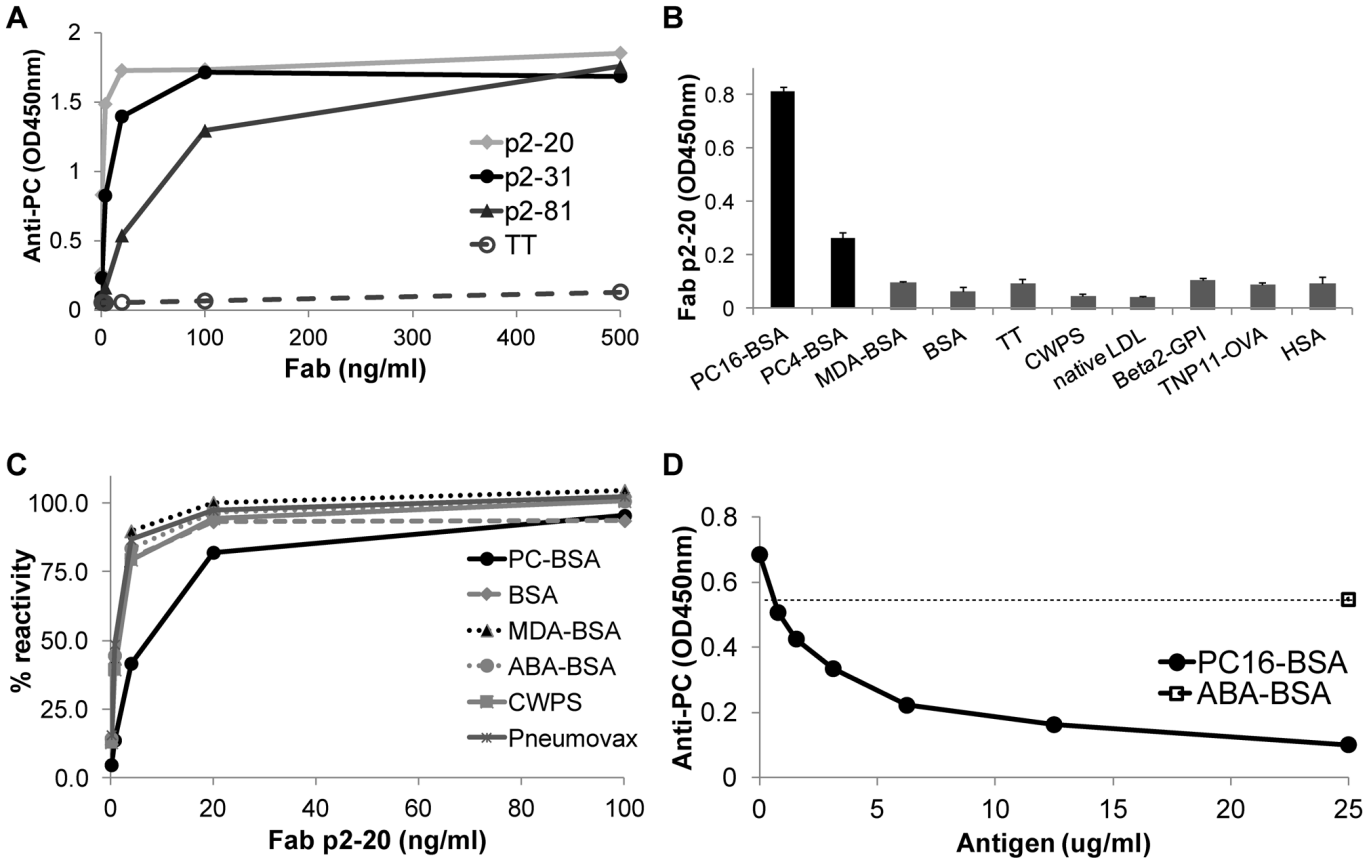
ELISA characterization of anti-PC purified Fab antibody fragments. ELISA binding of Fab purified antibody fragments. **A.** Binding of anti-PC clones p2–20, p2–31, and p2–81 to PC4-BSA at the indicated concentrations. Anti-tetanus toxoid (TT) Fab protein was used as control. **B.** Binding of Fab clone p2–20 at 500 ng/ml to PC16-BSA or PC4-BSA compared to the control antigens MDA-BSA, BSA, tetanus toxoid (TT), pneumococcal cell wall polysaccharide (CWPS), native human LDL, human β2-GPI, TNP conjugated ovalbumin (TNP11-OVA), or human serum albumin (HSA). **C.** ELISA binding to PC4-BSA of purified Fab at the indicated concentrations in the presence of different blocking antigens at 10 µg/ml. **D.** ELISA showing binding to PC4-BSA of the purified Fab anti-PC clone, p2–20, at 10 ng/ml in the presence of the indicated concentrations of PC16-BSA or ABA-BSA blocking antigen.

Fab clones p2–20, p2–31, and p2–81 were then expressed and purified as soluble Fab antibody fragments. While p2–7 was found to have an antibody expression level that was inadequate for further studies, the purified antibody fragments from the other three clones all exhibited substantial binding activity for PC-BSA (Figure 3), but not to a panel of structurally unrelated antigens. While clone p2–81 had somewhat lower binding activity in ELISA, p2–20 and p2–31 had similar binding reactivity and specificity (Figure 3 and data not shown). Importantly, direct PC-binding activity could be specifically inhibited by soluble PC-BSA in a dose-dependent manner. Interesting, despite significant binding activity for the PC-BSA conjugate, we found no evidence of binding to pneumococcal cell wall polysaccharide (CWPS, Figure 3) or PC-salt (data not shown). Hence the isolated clones showed a fine binding specificity akin to previously described murine B-cell hybridoma clones isolated after in vivo PC-KLH immunization, which have been shown to bind to protein conjugated PC as well as to neo-determinants on AC, but without cross-reactivity with CWPS-PC epitopes [9], [32]. Studies of murine immune responses have shown that these types of anti-PC antibodies more commonly arise in T-cell dependent responses [33], however the fine binding specificity of human antibodies to different classes of PC-containing antigens on ACs has not been as extensively described.

**Figure 4:**
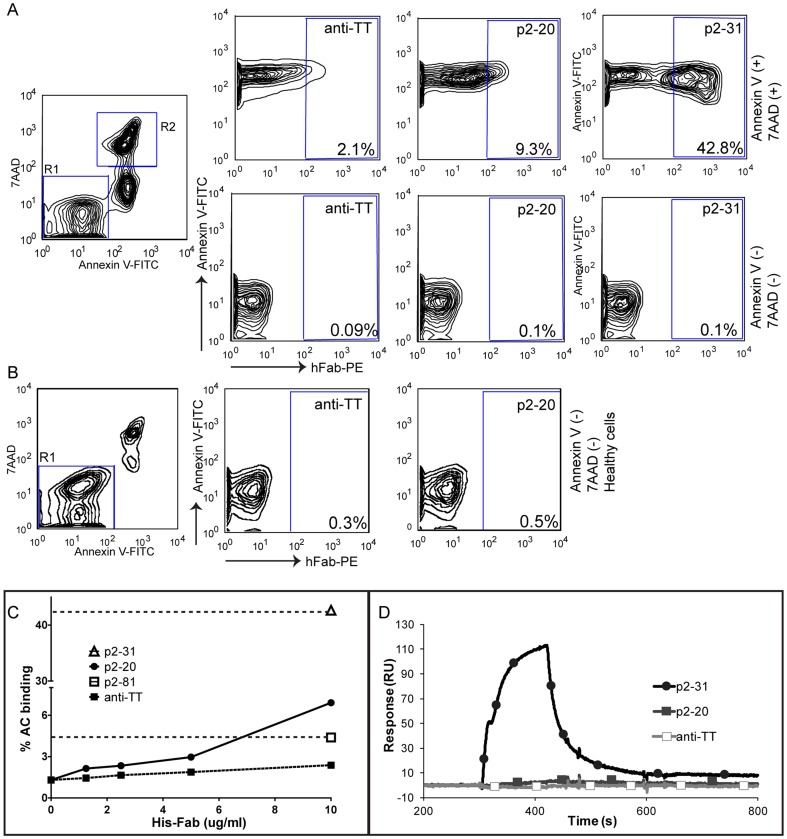
Flow cytometry and biosensor characterization of purified Fab antibody fragments. **A**. Flow cytometry analysis of purified Fab proteins binding to apoptotic murine thymocytes treated with 1 µM dexamethasone for 4 hrs. Left panels show the distribution of Annexin V and 7AAD positive cells. The top panel show binding to Annexin V(+) 7AAD(+) ACs (gate R2) of the control anti-TT Fab fragment, or anti-PC Fab clones p2–20 and p2–31 at 10 µg/ml, detected with anti-human Fab-PE. The Fab proteins did not bind to Annexin V (−) 7AAD (−) cells (gate R1) shown in the bottom panel. **B.** Flow cytometry binding of Fab proteins to non-treated freshly isolated thymocytes. **B.** Dose-dependent flow cytometry binding of the anti-PC Fab clone 2–20 to Annexin V (+) 7AAD (+) apoptotic cells compared to the control anti-TT Fab, at the indicated concentrations. In addition, anti-PC Fab clones 2–31 and 2–81 were analyzed at one concentration, 10 µg/ml. **C.** Biacore binding of Fab proteins to PC-containing ligand. Sensorgrams are shown for Fab antibody fragments injected separately at 5 µg/ml over immobilized PC4-BSA.

All tested purified anti-PC Fab antibody fragments showed specific binding to apoptotic but not to healthy cells (Figure 4). While the anti-PC clones, p2–31 and p2–20, had similar reactivity in ELISA assays, flow cytometric analyses using purified antibodies revealed higher relative binding reactivity by the more hypermutated VH1-encoded Fab, p2–31. Similarly, analyses of the binding interaction of Fab to PC4-BSA by surface plasmon resonance based assays showed that p2–31 had a significantly higher binding signal at 100 nM than was detected for the germline-configuration p2–20 (Figure 4). None of the analyzed Fab fragments interacted with the control surface and they had comparable binding to an anti-human Fab surface (data not shown). In summary, the selected human antibodies displayed binding activity for both the PC-protein conjugate and for the surface of ACs in a dose-dependent and antigen-inhibitable fashion.

### Human recombinant IgM anti-PC binding to ACs

**Figure 5:**
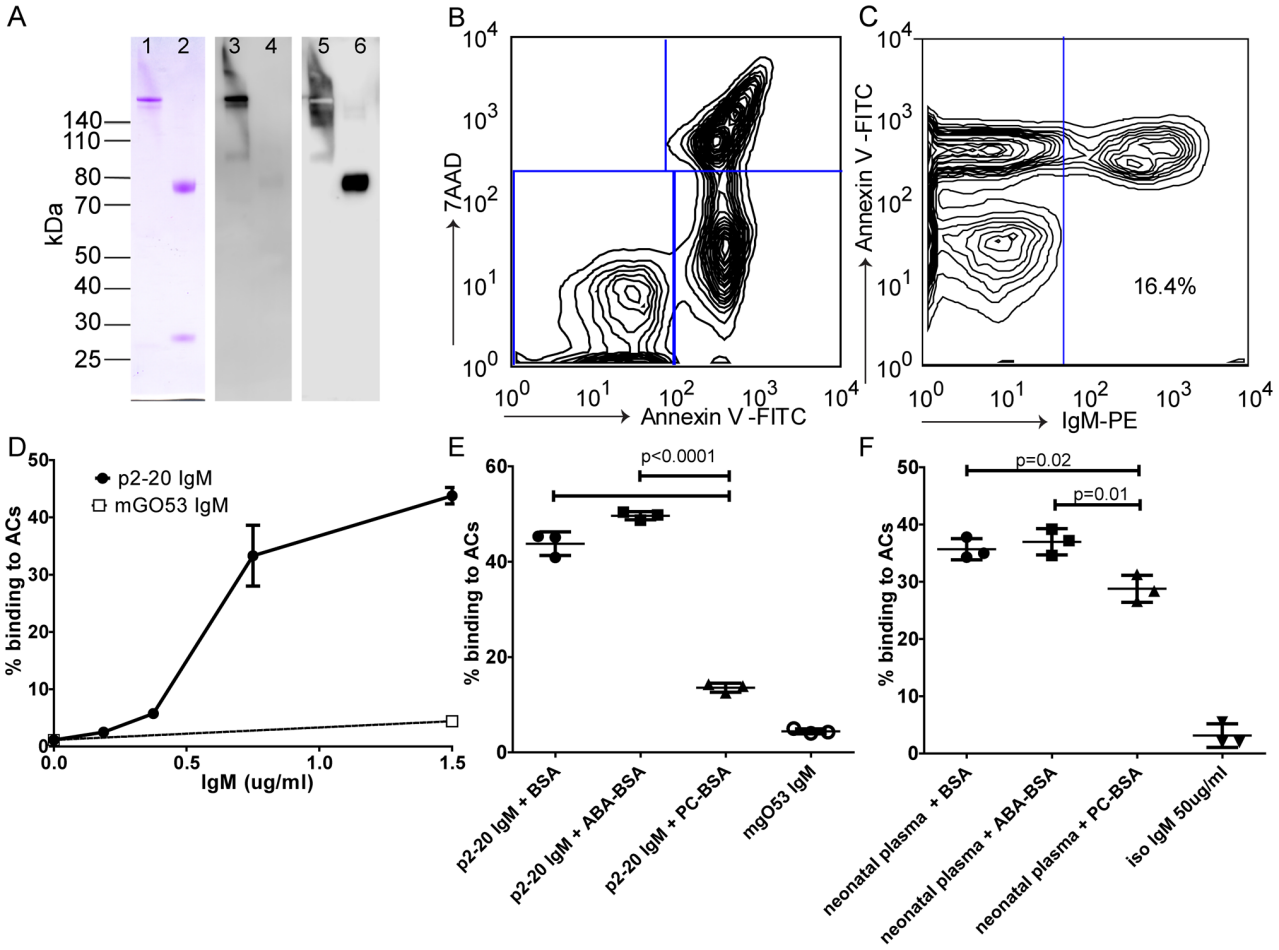
Binding of recombinant human IgM anti-PC to ACs. **A.** SDS-PAGE of Fab-mediated protein A purified IgM anti-PC clone p2–20 for 1 µg non-reduced (1, 3, 5) or reduced antibody (2, 4, 6). Lanes 1-2 show Coomassie brilliant blue staining, lanes 3-4 show an immunoblot staining with a conformational sensitive anti-human IgM μ-chain specific antibody (Southern Biotech) and lanes 5-6 show the membrane re-probed with a goat polyclonal anti-human IgM μ-chain specific antibody (Jackson ImmunoResearch). **B.** Flow cytometry distribution of 7AAD and Annexin V positive thymocytes after induction of apoptosis by 4 hrs incubation with dexamethasone. **C**. Analysis of the binding of IgM anti-PC clone p2–20 without sub-gating. IgM crude expression supernatant were diluted 1∶2 in 3% BSA in PBS and assessed for apoptotic cell binding. IgM p2–20 only binds to the 7AAD (+) Annexin V (+) AC population. **D.** Dose-dependent binding of IgM p2–20 to Annexin V (+) 7AAD (+) apoptotic thymocytes compared to the isotype control IgM mGO53. Binding of IgM p2–20 supernatants (**E**) or neonatal plasma pool of 4 healthy babies at 1∶25 dilution (**F**) to 7AAD (+) Annexin V (+) AC in the presence of BSA, ABA-BSA, or PC16-BSA antigens at 100 µg/ml. Binding was compared to purified isotype control myeloma IgM (Jackson ImmunoResearch) at 50 µg/ml or recombinant isotype control IgM (mGO53) at 4.5 µg/ml.

Since natural antibodies that recognize ACs are typically of the IgM subclass, we subcloned and expressed the abundant anti-PC clone, p2–20, as a recombinant IgM (Figure 5A), using previously described methods [24]. Expression levels in the HEK293T system were generally ∼ 2 µg/ml for p2–20 and 9 µg/ml for the control IgM, mGO53, as determined by μ-chain specific ELISA. Importantly, while the expressed p2–20 IgM showed strong binding to 7AAD (+) Annexin V (+) apoptotic murine thymocytes, we did not detect any binding to Annexin V negative cells (Figure 4B and C). Furthermore the AC binding was dose-dependent and inhibitable by soluble PC-BSA antigen but not by control antigens (Figure 4 D–F). Indeed, binding was reduced by 69% by PC-BSA blockade (13.6±0.95% binding) compared to incubation with non-conjugated BSA (43.8±2.5% binding) (Figure 4E). The control IgM expressed in the same system did not show detectable binding in these cellular assays. Moreover, the physiologic relevance of these results was further documented when we found that polyclonal IgM antibodies in human umbilical cord plasma (from a newborn) also bound to 7AAD (+) Annexin V (+) ACs, and that this binding was significantly reduced (overall ∼18%) by PC-BSA antigen blockade (28.8±2.4% binding) compared to incubation with BSA alone (35.7±1.9% binding) (Figure 5F). These findings are therefore consistent with evidence that PC-specific natural IgM antibodies contribute to AC-binding in humans at birth. Hence, future studies are merited to further evaluate the relative representation of anti-PC antibodies in neonates compared to adults and their possible relationship to the herein described human anti-apoptotic cell monoclonal antibodies that we isolated from adult bone marrow.

### Molecular modeling of potential antigen binding sites

The antigen binding site in antibodies is classically formed by contacts that can be associated with side chains from the three H chain CDRs and the three L chain CDRs, which together generate a composite surface [34]. The HCDR3 commonly resides in a central position of this surface [34] and the contribution of HCDR3 alone has been shown in many cases to be sufficient to generate an antigenic fine binding specificity [35]. The antigen binding site of an antibody can exhibit a great potential range of surface topologies, from a small binding recess or pit, as seen for the prototypic murine anti-PC McPC603 antibody (PDB ID: 2MCP), to narrow grooves or extended protruding surfaces, which can each define the nature of an antigen-antibody binding interaction.

**Figure 6:**
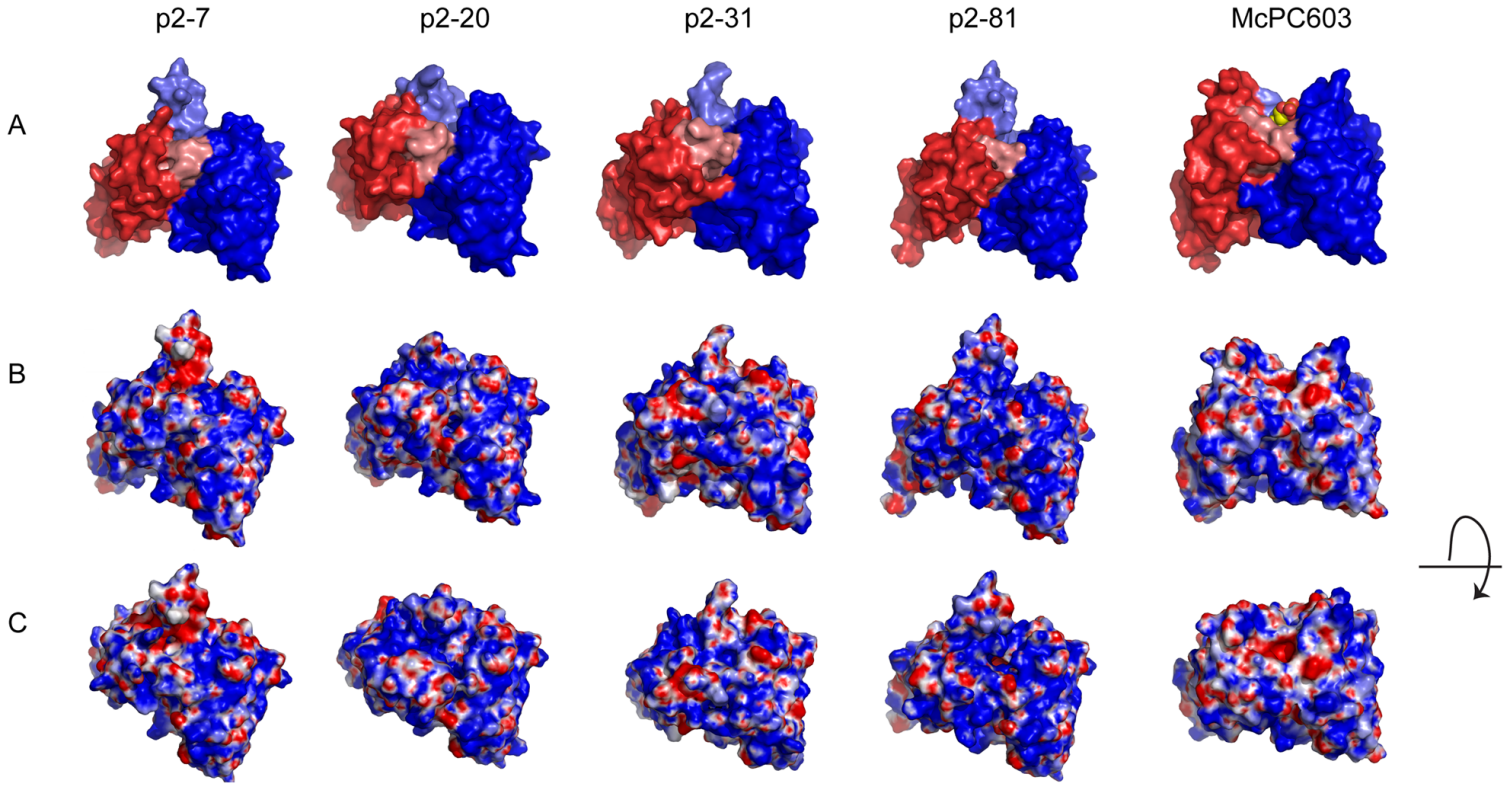
Structure models of the variable region of the selected human anti-PC antibodies. Models are shown for the anti-PC clones: p2–7, encoded by a VH3-30 and Vκ3-20; p2–20, encoded by a VH3-33 and Vλ1-44; p2–31, encoded by a VH1-2 and Vλ1-47, and p2–81, encoded by VH3-30 and Vκ1-5 rearrangements. The models are compared to the crystal structure of the PC-binding murine antibody McPC603, with the PC-antigen in the binding pocket (PDB ID: 2MCP). **A**. The VL region is visualized in red and the VH region in blue. HCDR3 is highlighted in lighter blue shade and LCDR3 in lighter red shade. **B.** Electrostatic surface models of the variable regions. Blue color represents positively charged surface residues and red negatively charged residues. **C.** Electrostatic surface models with a view looking into the potential antigen binding site. Models of the variable region surfaces were generated by WAM tool [28], using dead-end elimination for the side-chains building and VFF energy screen method. Illustrations of the structures were generated in PyMol (DeLano Scientific LLC) using APBS for electrostatic calculations [30].

To investigate the surface topologies associated with the potential antigen binding sites of the selected human anti-PC antibodies, we performed structural modeling studies for visualization of the potential surfaces generated by their CDRs (Figure 6). As anticipated, these models demonstrated similarities between the VH3-30 gene encoded p2–7 and p2–81 clones, which have long HCDR3s that were predicted to represent loops that project out from the potential antigen-binding site. In contrast, clones p2–20 and p2–31, which have HCDR3 lengths closest to the mean length reported for peripheral mature B cells of healthy adults [31], [36], were predicted to have more conventional features (Figure 6). Notably, while anti-PC responses in immunocompetent murine strains are reported to be commonly dominated by T15 idiotypically related B-cell clonal sets that use short related HCDR3 rearrangements of the S107.1 VH gene, none of the selected human anti-PC clones showed significant homology in sequence or in length with known HCDR3 of T15 related antibodies (data not shown). Interestingly, the different human anti-PC clones showed a variety of predicted surface structures, ranging from extended HCDR3 loops in p2–7 and p2–81 to a more groove-like structure in p2–20, while p2–31 had a variable region encoded surface that appeared to have a small potential binding pit. Calculations of electrostatic charges for the modeled surfaces revealed mostly positive charges in the center of the composite surface generated by these CDR loops (Figure 6).

## Discussion

To investigate the immunogenetic and structural diversity among human antibodies that recognize ACs, we have used phage display technology to select antibodies that specifically bind to PC-containing ligands and that can discriminate apoptotic from healthy cells. We were surprised to find that three of the four dominant selected Fab clones used germline or near germline configuration VH regions. Moreover, the long HCDR3 in clones p2–7 and p2–81 are reminiscent of reported antibodies with polyreactive properties [37]. In addition, we also recovered the p2–31 clone that uses a more hypermutated VH gene rearrangement, which may indicate that it originated from a T-cell dependent germinal center response. It is possible that the VH region from this clone came from a different in vivo cellular source than the (near) germline VH regions of the other three clones. This may also explain the differences in binding affinity both for the experimental selecting ligand (i.e., PC-BSA) antigen and for the surfaces of ACs. Nonetheless, when the dominant germline encoded clone p2–20 was expressed as recombinant IgM, which likely represents the original antibody isotype format for this germline configuration antibody, it showed impressive dose-dependent binding to ACs that was inhibitable by soluble PC antigen. Furthermore, natural IgM present at birth commonly display AC binding activity as demonstrated by our flow cytometric studies of IgM in human neonatal plasma (Figure 5). Hence, our studies provide evidence of the properties of human monoclonal anti-PC antibodies that specifically bind to ACs.

As an increasing number of reports collectively suggest that the natural IgM autoantibodies that recognize epitopes on ACs, in the context of Fcμ domains, have innate-like properties and play critical roles in maintaining homeostasis. In mice, germline-encoded IgM natural autoantibodies that bind to the PC head group in oxidized lipids have the ability to recruit the complement recognition factors, C1q and mannose binding lectin (MBL), to AC membranes and enhance phagocytic clearance by innate cells while also inducing potent anti-inflammatory signaling pathways. The murine B1-derived prototypic anti-PC antibody, T15, and idiotypically related antibodies, have been extensively studied and these can express non-hypermutated canonical variable gene rearrangements and arise spontaneously early in life [38], [39]. Indeed, in mice, circulating natural IgM are predominantly secreted by B-1 cells [40], which have a specialized phenotype [41] and the capacity to be recruited into T-cell independent responses to non-protein antigens (reviewed in [42]). Furthermore, the murine B-1 cell repertoire has been associated with an increased preference of antibodies encoded by germline variable gene segment without evidence of somatic hypermutations [43]. Murine B-1 clones also more frequently have gene rearrangements without N-insertions in the V-D and D-J junctions [44] suggesting that they arise before birth in the fetal liver that lacks expression of the terminal deoxynucleotidyl transferase (TdT) required for this diversification mechanism. Notably, murine IgM anti-PC have been demonstrated to inhibit toll-like receptor (TLR)-mediated inflammation [45]–[47], suppress disease in murine models of inflammatory arthritis [45] and halt the progression of atherosclerosis in hypercholesterolemic mice [48]. In humans, recent clinical surveys have demonstrated that higher levels of IgM anti-PC are associated with less active disease in SLE and protection from cardiovascular disease in both SLE and non-autoimmune patients [12], [13], [49]–[51].

Significantly, three out of four unique human antibody clones identified in the selection were encoded by germline configuration VH region genes with no somatic hypermutations in the CDR regions. Yet, these human clones all display N-insertions. However, TdT is expressed in humans throughout immune development [52] and hence our antibody sequence analysis could not provide insights into cellular origins. Furthermore, the limitations of the technical approach used in the current studies did not enable the identification of the antibody isotype of the parental clone, as antibodies recovered by phage display may or may not maintain the in vivo H-L chain pairing [53]. Thus, the L chains used in the selected human antibodies, which uniformly showed extensive hypermutation, may not represent the original L chain pairing. Nevertheless, the L chains may also contribute to the contacts of the antigen-binding site with the antigen. Despite the limitations, there are reports in which phage display has enabled recovery of antibodies with VH-VL usage that mirror dominant in vivo clonal representation [53], [54].

While the existence and properties of human B-1 cells have long been controversial topics, in a recent report human B-1 cells have been described with a CD27+ CD43+ CD70- phenotype. This B-cell subset has been detected both in human umbilical cord and adult blood and was found to spontaneously express higher levels of IgM anti-PC than other subsets [55]. The potential relationship between these B cells and anti-PC antibodies we herein describe will merit further investigation.

In the current study, we found antibodies that bound PC conjugated to a protein carrier but not to CWPS. While there can be different PC epitopes in these different antigens, these epitopes may display some structural differences. In the mouse, two classes of PC binding profiles have been suggested based on the ability (or inability) to cross-react with CWPS [9]. However, there is currently only limited evidence that this is also true in humans, and early studies have shown there be even greater greater heterogeneity in the PC reactivity of antibodies in humans than in mice [11], [56], [57]. Yet, a recent study of human responses to the PC containing vaccine Pneumovax revealed a preferential usage of VH3-30 and VH3-33 encoded regions in anti-CWPS responses, which may suggest some shared structure-function relationships with the currently described anti-PC clones; p2–7, p2–20 and p2–81. However, in contrast to the selected VH3-encoded anti-AC antibodies that had little evidence of hypermutation, these previously reported anti-CWPS antibodies, which were recovered by the sorting of single B cells in post-immunization individuals, were found to instead display high levels of somatic hypermutation [58]. Furthermore, we also could not detect binding reactivity of the dominant clone, p2–20, to oxidized LDL that is known to express PC-containing epitopes (data not shown).

Future studies will therefore be required to further characterize the antigen-binding capacities of the monoclonal human anti-PC antibodies, and to evaluate alternative phage display selection strategies. It would be especially interesting to compare the currently described antibodies to the antibodies expressed by B-cells in neonates. In addition, future cDNA libraries can be restricted to only include transcripts from IgM-expressing B-cells in bone marrow or by combining technical approaches with cell sorting efforts, which can be used to recover antibodies expressed by distinct B-cell subsets. Furthermore, recent technical advances for high-throughput cloning of paired H-L chain B cell repertoires could also be applied to further characterize the in vivo anti-PC response [59], [60]. Hence, while the current study may not elucidate the complete diversity among human anti-PC antibody clones, and our current studies of isolated human monoclonal antibodies provide an essential step towards understanding the structural and genetic bases for this prominent autoreactive binding system.

Based on recently reported murine models of inflammation and autoimmunity, there may be potential therapeutic applications for human anti-PC antibodies [45], [48]. In conclusion, advances in our understanding of the immunogenetic and structural features of homeostatic natural antibodies in humans may provide diagnostic, prognostic, and therapeutic opportunities.

## Supporting Information

Table S1 (PDF)
