# Supplementary material for: Selection of Apoptotic Cell Specific Human Antibodies from Adult Bone Marrow
Source: PLoS One. 2014 Apr 23;9(4):e95999. doi: 10.1371/journal.pone.0095999 (PMC3997490; doi:10.1371/journal.pone.0095999)
Supplement: Table S1 — (PDF) [file pone.0095999.s001.pdf]

Table S1. VL sequences of PC-binding clones from phage display selection compared to the closest germline sequence

	FWR1	CDR1	FWR2	CDR2	FWR3			J
**p2-7**	LTQSPGPLSLSPGERVTLSCRAS	QSVRS-D	LAWYQQRPGQPPRLLIY	DAS	IRAIGIPDRFSGSGSGTDFTLTISRVESEDFAVYYC	QQYGGS	F	LTFGPGTKVD
VK3-20*01	******T********A*******	***S*SY	******K***A******	G**	S****T*******************L*P********	****S*		
JK3*1								**********
**p2-20**	QPVLTQPPSASGAPGQRVTISCSGS	SSNIGTYS	VNWYQQLPGTAPKLLIY	RNN	QRPSGVPDRFSGSKSGTSAALAISGLQSEDEADYYC	ATWDGSLHG		WVFGGGTKLTVL
VL1-47*01	*S**********T************	******NT	**************	S**	*******************S****************	*A**D**N*		
JL3*2								************
**p2-81**	IRLTQSPSTLSAFVGDRVTITCRAS	QSINNW	LAWYQQKPGRAPKALIY	KAS	TLESGVPSRFSGSGSGTEFTLTITNLQPDDFATYYC	QQYNGDS	RA	FGQGTKVEIK
VK1-5*01	*QM*********S************	***SS*	*********K***L***	***	S**********************SS***********	*****Y*		
JK1*01								**L******
**p2-31**	ELTQPLSTSGTPGQRVSISCSGS	SSNIGSNF	VYWFQQFPGTAPKLLIY	RNN	RRPSGVSDRFSGSRSGTSASLAISGLRSVDEADYYC	ASWDDNLS	A	YVFGTGTKVTVL
VL1-47*01	V****P*A*********T*****	*******Y	***Y***********	***	Q*****P******K**************E******	*A***S*		
JL1*01								************

* Identical to the closest known VL/JL germline gene
